# Supplementary material for: SERS Studies of Adsorption on Gold Surfaces of Mononucleotides with Attached Hexanethiol Moiety: Comparison with Selected Single-Stranded Thiolated DNA Fragments
Source: Molecules. 2019 Oct 30;24(21):3921. doi: 10.3390/molecules24213921 (PMC6864626; doi:10.3390/molecules24213921)
Supplement: Supplementary file 1 [file molecules-24-03921-s001.pdf]

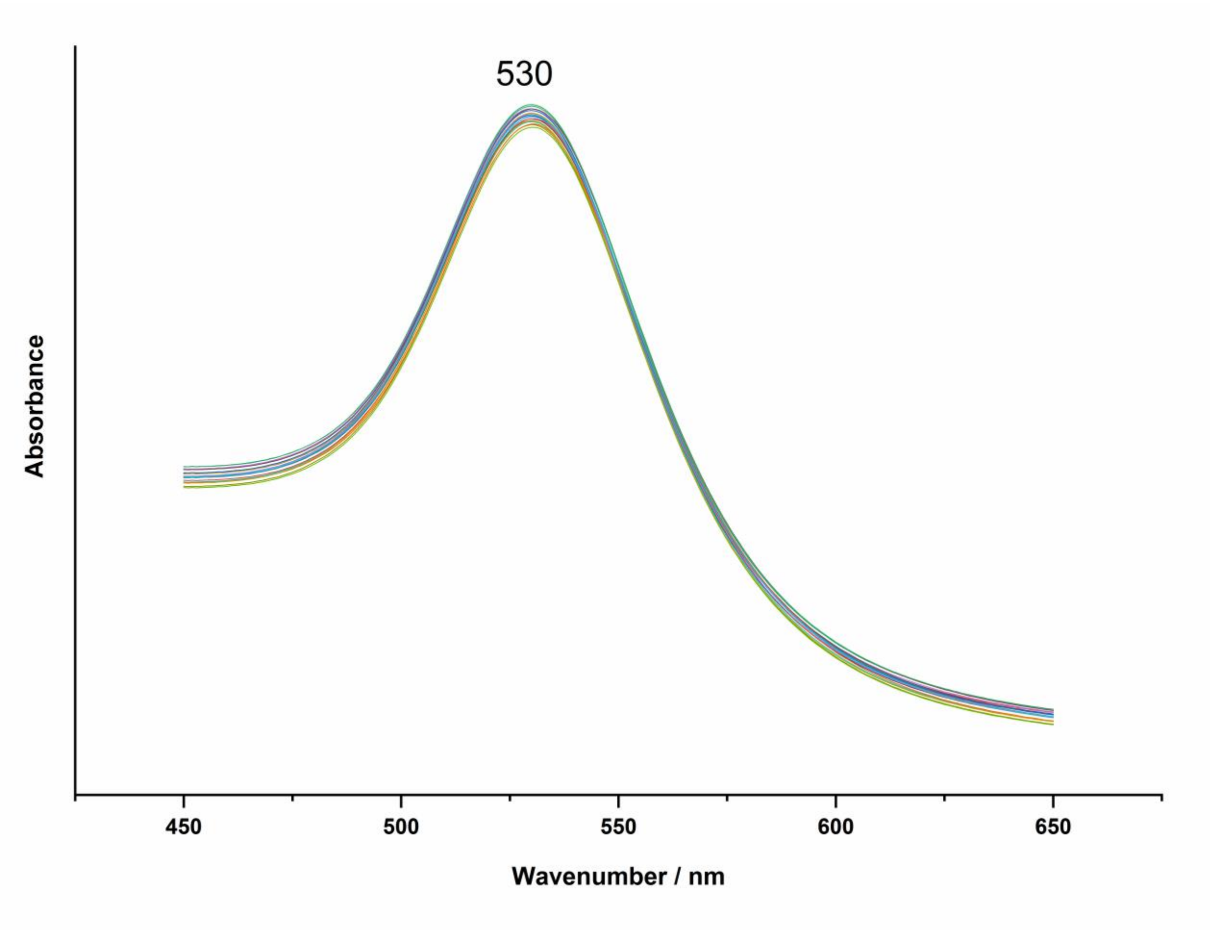

Figure S1. UV-Vis spectra of nanoparticles and nanoparticles with 18 molecules studied in the manuscript: , adenine, cytosine, guanine, thymine and ssDNA: A-$(CH_2)_6$-SH, C-$(CH_2)_6$-SH, G-$(CH_2)_6$-SH, T-$(CH_2)_6$-SH, CA-$(CH_2)_6$-SH, C_3A-$(CH_2)_6$-SH, C_7A-$(CH_2)_6$-SH, C_{11}A-$(CH_2)_6$-SH, C_{15}A-$(CH_2)_6$-SH, AC_{15}-$(CH_2)_6$-SH, C_7AC_8-$(CH_2)_6$-SH, T_3A-$(CH_2)_6$-SH, T_7A-$(CH_2)_6$-SH, and T_{11}A-$(CH_2)_6$-SH.

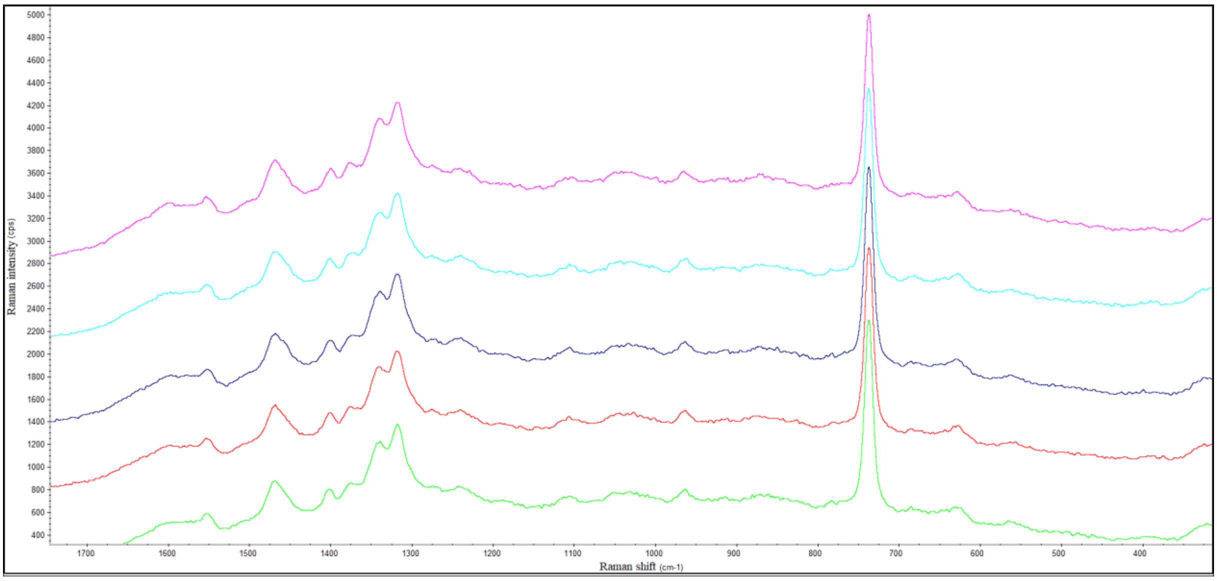

Figure S2. SERS spectra of adenine thiolated mononucleotide collected on one sample in different places. The difference in spectral intensity is around 5%.

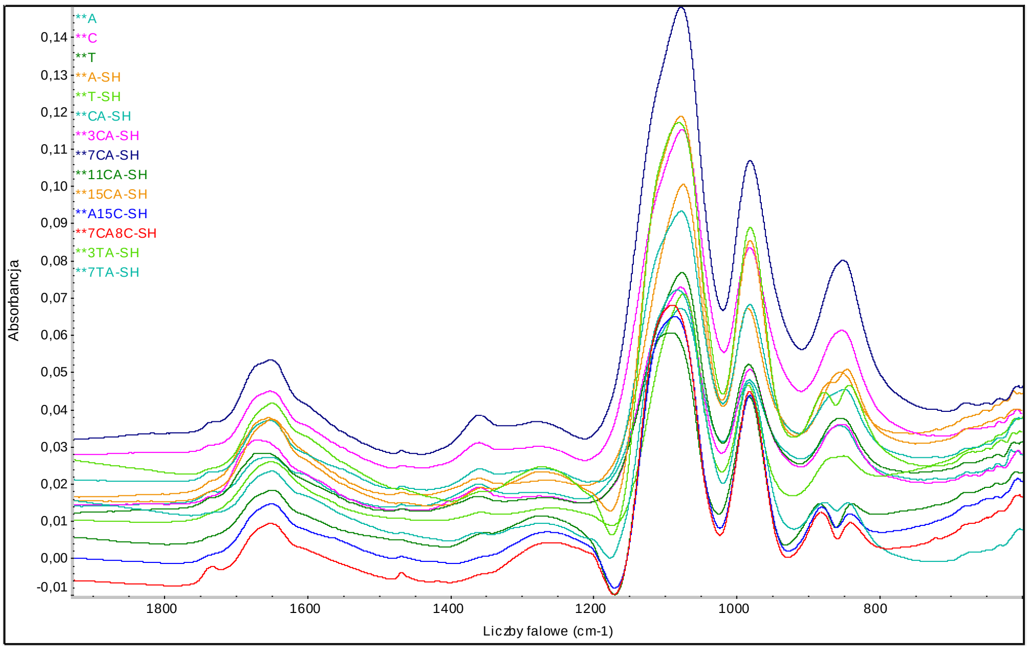

Figure S3. FT-IR spectra of selected samples measured previously by SERS: adenine, cytosine, thymine, A-$(CH_2)_6$-SH, T-$(CH_2)_6$-SH, CA-$(CH_2)_6$-SH, C_3A-$(CH_2)_6$-SH, C_7A-$(CH_2)_6$-SH, C_{11}A-$(CH_2)_6$-SH, C_{15}A-$(CH_2)_6$-SH, AC_{15}-$(CH_2)_6$-SH, C_7AC_8-$(CH_2)_6$-SH, T_3A-$(CH_2)_6$-SH and T_7A-$(CH_2)_6$-SH.
